# Variations and implications of Irisin, FGF-23, and N-MID osteocalcin in diabetic osteoporosis patients undergoing acarbose plus sitagliptin therapy: A prospective cohort analysis

**DOI:** 10.5937/jomb0-60451

**Published:** 2026-03-17

**Authors:** Lilan Zhou, Linghua Peng

**Affiliations:** 1 Affiliated Hospital of JiuJiang University, Department of Endocrinology, Jiujiang, China; 2 The First Affiliated Hospital of Gannan Medical University, Department of Burn and Wound Repair, Gannan, China

**Keywords:** diabetic osteoporosis, Irisin, FGF-23, N-MID osteocalcin, biomarkers, dijabetička osteoporoza, Irizin, FGF-23, N-MID osteokalcin, biomarkeri

## Abstract

**Background:**

The present study intends to explore the dynamic variations in Irisin, fibroblast growth factor 23 (FGF-23), and N-terminal propeptide of osteocalcin (N-MID) among diabetes mellitus osteoporosis (DOP) patients undergoing treatment with acarbose plus sitagliptin, as well as their ability to predict bone metabolic status and fracture risk. The ultimate objective is to identify potential biomarkers for optimizing DOP therapy.

**Methods:**

From January 2023 to March 2024, 124 DOP patients admitted to our hospital were administered acarbose in combination with sitagliptin. For comparison, 119 uncomplicated diabetes mellitus (DM) patients were recruited as controls. Serum levels of Irisin, FGF-23, and N-MID were tested both at baseline and post-treatment. Data on bone mineral density (BMD), bone metabolic markers, and fracture occurrences during follow-up were also collected.

**Results:**

Compared with uncomplicated DM individuals, DOP patients exhibited a rise in FGF-23 (P&lt; 0.05) and a decrease in Irisin and N-MID (P&lt; 0.05). Irisin + FGF-23 + N-MID combination demonstrated 87.10% sensitivity and 74.58% specificity in diagnosing DOP (AU C= 0.867). Treatment with acarbose and sitagliptin contributed to reduced FGF-23 in DOP patients, along with increased Irisin and N-MID (P&lt; 0.05). BMD was positively influenced by irisin but negatively affected by FGF-23. Fracture prediction efficacy was markedly enhanced (AU C= 0.822) by combining the three indices, with statistical superiority over single-index analysis (P&lt; 0.05).

**Conclusions:**

The combined detection of Irisin, FGF-23, and N-MID enables early detection of DOP while allowing for dynamic assessment of treatment outcomes and fracture risk.

## Abstract (sr)

**Uvod:**

Ova studija ima za cilj da istraži dinamičke varijacije irisina, faktora rasta fibroblasta 23 (FGF-23) i N-terminalnog propeptida osteokalcina (N-MID) kod pacijenata sa dijabetes melitusom i osteoporozom (DOP) koji se leče akarbozom plus sitagliptinom, kao i njihovu sposobnost da predvide status metabolizma kostiju i rizik od preloma. Krajnji cilj je identifikacija potencijalnih biomarkera za optimizaciju DOP terapije.

**Metode:**

Od januara 2023. do marta 2024. godine, 124 pacijenta sa DOP primljenih u našu bolnicu primali su akarbozu u kombinaciji sa sitagliptinom. Poređenja radi, 119 pacijenata sa neusložnjenim dijabetes melitusom (DM) regrutovano je kao kontrolna grupa. Serumski nivoi Irisina, FGF-23 i N-MID testirani su i na početku i nakon lečenja. Takođe su prikupljeni podaci o mineralnoj gustini kostiju (BMD), markerima metabolizma kostiju i pojavi preloma tokom praćenja.

**Rezultati:**

U poređenju sa osobama sa neusložnjenim dijabetesom, pacijenti sa DOP pokazali su porast FGF-23 (P&lt; 0,05) i smanjenje irisina i N-MID (P&lt; 0,05). Kombinacija Irisin + FGF-23 + N-MID pokazala je osetljivost od 87,10% i specifičnost od 74,58% u dijagnostikovanju DOP (AU C= 0,867). Lečenje akarbozom i sitagliptinom doprinelo je smanjenju FGF-23 kod pacijenata sa DOP zajedno sa povećanjem irisina i N-MID (P&lt; 0,05).

**Zaključak:**

Kombinovana detekcija Irisina, FGF-23 i N-MID omogućava rano otkrivanje DOP-a, a istovremeno omogućava dinamičku procenu ishoda lečenja i rizika od preloma.

## Introduction

A concurrent condition of diabetes mellitus (DM) and osteoporosis (OP) is known as diabetes mellitus osteoporosis (DOP). Its pathological mechanisms are both intricate and distinctive, involving a range of contributing factors like hyperglycemic toxicity, insulin resistance, persistent inflammation, and oxidative stress [1]. Not only does this condition directly impede osteoblast activity and boost osteoclast activation, but it also meddles with the muscle-bone axis regulation, disturbs phosphorus metabolism equilibrium, and impairs bone formation-mineralization coupling. Such disruptions lead to an uneven bone turnover rate, greatly increasing fracture susceptibility [2]. Epidemiological data indicate that roughly 30% of global DM individuals suffer from osteopenia or OP [3]. With DOP constituting a major obstacle in managing diabetes-related chronic complications, elucidating core modulators of dysregulated bone metabolism and assessing intervention outcomes early has become essential strategies to minimize fracture susceptibility [4].

Existing studies predominantly focus on bone mineral density (BMD) variations or short-term evaluation of individual bone metabolism markers in DOP patients, resulting in limited clinical insights into disease evaluation and prediction in DOP [5][6]. Our investigation therefore incorporates Irisin (muscle-derived signaling), fibroblast growth factor 23 (FGF-23) (phosphate homeostasis regulator), and N-terminal propeptide of osteocalcin (N-MID) (osteogenic activity marker), biomarkers that collectively capture DOP's core pathological mechanisms, while accounting for their complex interrelationships. Specifically, irisin, as a critical mediator of the muscle-bone axis, is produced by skeletal muscle and drives bone formation through osteoblast differentiation activation, with its secretion directly dependent on muscle contractions (such as during exercise) and hyperglycemia [7]. FGF-23, secreted by bone cells, is the core regulator of phosphorus metabolism. Its increase inhibits 1α-hydroxylase activity, lower active vitamin D production, and hinder intestinal calcium absorption, thus inducing bone mineralization disorders [8]. As the N-terminal propeptide of type I collagen (PINP), N-MID is a clinically validated early indicator of osteogenic activity, where circulating levels quantitatively reflect osteoblast performance [9]. In DOP patients, Irisin may influence N-MID secretion by modulating osteoblast functioning through the muscle-bone axis; FGF-23 may impair N-MID-mediated bone formation by interfering with phosphorus metabolism and vitamin D activation.

The above evidence suggests that tracking irisin, FGF-23, and N-MID variations in DOP could yield innovative biomarkers for clinical assessment. Therefore, this study analyzes changes and clinical implications of Irisin, FGF-23, and N-MID during DOP treatment, so as to provide multi-dimensional biomarker targets for tailored DOP treatment strategies.

## Materials and methods

### Research participant selection

Conducted as a prospective cohort analysis, this research focuses on DOP patients hospitalized at our institution between January 2023 and March 2024. Prior to commencement, the study obtained formal ethical clearance from our hospital's Institutional Review Board, with all procedures conforming strictly to Helsinki Declaration standards.

### Eligibility and exclusion criteria

Eligibility criteria: Individuals aged 65 or above, irrespective of sex, were enrolled if they satisfied DM [10] and OP [11] diagnostic criteria. Besides, intact clinical documentation and participant consent were obligatory.

Exclusion criteria: Participants were disqualified for: severe hyperglycemia or hypoglycemia (HbA1c levels <7.5% or >10%); comorbid disorders influencing bone turnover (e.g., hyperthyroidism, hypothyroidism, or rheumatoid arthritis); administration of bone-modifying agents in the prior quarter; significant impairment of liver/kidney function; history of malignancy; chronic alcohol intake (>20 g/day) or substance dependence.

### Sample size calculation and grouping

Using PASS software, the minimum sample size was determined with an α level of 0.05, [β = 0.90, effect size d = 0.5, based on pilot study data, incorporating a 20% dropout rate. The final cohort comprised 124 DOP patients and 118 uncomplicated DM patients who were hospitalized concurrently.

### Treatment methods

Upon admission, DOP patients were prescribed acarbose (50 mg/tablet, Bayer Pharmaceuticals) three times daily (chewed with the first bite of meals), along with sitagliptin (100 mg/tablet, Merck) given as a 100 mg once-daily dose (orally before breakfast).

### BMD testing

For BMD assessment, the lumbar spine (L1-L4) was scanned while the patient remained in a supine position. The scan duration ranged from 1 to 5 minutes, during which motionlessness was required.

### Laboratory examinations

Fasting venous blood (4 mL) was drawn from DM patients upon hospital admission and from DOP patients at both admission and post-treatment (4 weeks). Samples were collected into coagulation-promoting tubes, allowed to clot for 30 minutes at ambient temperature, then processed by centrifugation. The isolated serum was preserved at -80°C until analysis (all blood samples are completed at 8-10 a.m.).

Enzyme-linked immunosorbent assay detection of Irisin (Wuhan Baiyixin Biotechnology Co., LTD., Item number: TD711334) and FGF-23 (Beijing Boaosen Biotechnology Co., LTD., Product number: bsk11123): Each well was coated with 50 μL of either standard or sample, combined with 50 μL antibody solution, and incubated (37 °C, 2h). Following TMB substrate addition and 15-minute color development in darkness, reactions were stopped with sulfuric acid. A microplate reader read the optical density (450 nm). Sensitivities: Irisin 0.2 ng/mL (intra-assay coefficient of variation [CV]<8%, inter-assay CV< 10%); FGF-23: 3 pg/mL with linear range 3-2000 pg/mL.

Automated immunoassay analysis for N-MID, P1NP and β-crosslaps (β-CTX): Samples/calibrators were loaded onto testing modules (UniCelTM Dxl800 Access, Beckman Coulter, USA). The system automatically performed pipetting, incubation (37°C, 18 min), magnetic bead separation, and luminescent signal detection. Sensitivity thresholds: N-MID: 5 μg/mL; P1NP: 5 pg/L; [β-CTX: <0.010 μg/L.

Quality control: Daily runs of low/medium/high-level controls were conducted, analyzed using the Westgard multi-rule system (1_3_S/2_2_S/R_4_S).

### Follow-up for prognosis

All DOP cases underwent a follow-up lasting at least one year, conducted via regular clinical reviews. The follow-up period concluded in March 2025, with termination also triggered by the occurrence of an osteoporotic fracture in any patient (fractures due to low-energy trauma (falls from standing height), confirmed by X-ray/CT, and BMD T score ≤-2.5).

### Statistical methods

Statistical analyses were conducted with SPSS Version 25.0. Frequency data [n(%)] underwent χ^2^ analysis. Normally distributed measuring data (χ̄±s) were compared using either Student's t-test (independent or paired designs); non-normally distributed data [M (P25, P75)] required Mann-Whitney U or Kruskal-Wallis testing. Diagnostic accuracy assessments involved receiver operating characteristic (ROC) analysis with threshold optimization via Youden index maximization. Combination models derived from logistic regression equations underwent subsequent ROC evaluation. Pearson's correlation coefficients measured variable associations. A significance threshold of P<0.05 was adopted.

## Results

### Clinical data comparison

Patients' baseline data, shown in Table 1, revealed no statistical inter-group differences (P>0.05).

**Table 1:** Clinical data. Note: diabetes mellitus (DM), body mass index (BMI), fasting blood glucose (FBG), diabetes mellitus osteoporosis (DOP).

		DM (n = 119)	DOP (n = 124)	Statistical (t or χ^2^)	P
Age (years old), (χ̄±s)		70.74±3.86	71.09±4.15	0.679	0.498
Gender, [n (%)]	male	47 (39.50%)	44 (35.48%)	0.417	0.518
	female	72 (60.50%)	80 (64.52%)		
Duration of DM (years), (χ̄±s)		12.01±4.77	11.71±3.56	0.555	0.580
History of fractures,<br>[n (%)]	yes	12 (10.08%)	22 (17.74%)	2.959	0.085
	no	107 (89.92%)	102 (82.26%)		
BMI (kg/m^2^), (χ̄±s)		25.59±3.20	25.89±1.98	0.905	0.367
Smoking, [n(%)]	yes	35 (29.41%)	46 (37.10%)	1.614	0.204
	no	84 (70.59%)	78 (62.90%)		
Drinking, [n (%)]	yes	26 (21.85%)	38 (30.65%)	2.422	0.120
	no	93 (78.15%)	86 (69.35%)		
Combined<br>hypertension, [n (%)]	yes	42 (35.29%)	48 (38.71%)	0.304	0.582
	no	77 (64.71%)	76 (61.29%)		
FPG (mmol/L), (χ̄±s)		13.77±3.75	14.07±4.11	0.601	0.549

### Expression and clinical significance of Irisin, FGF-25, and N-MID in DOP

**Figure 1:**
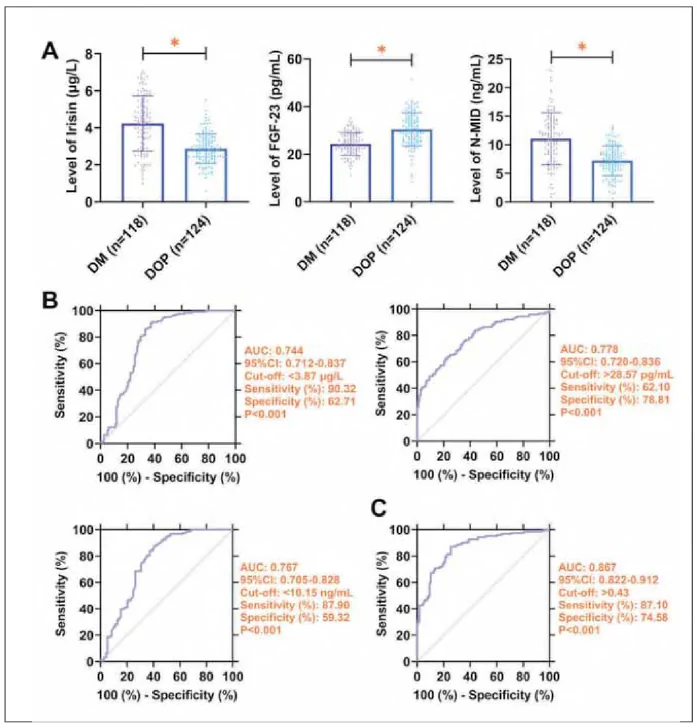
Diagnostic efficacy of Irisin, FGF-23, and N-MID for DOP. * indicates P < 0.05.(A) Comparison of Irisin, FGF-23, and N-MID levels between patients with DM and those with DOP. (B) Diagnostic effect of Irisin, FGF-23 and N-MID on DOP in patients with DM.(C) The diagnostic effect of combined detection of Irisin, FGF-23 and N-MID on DOP in patients with DM.

Compared with uncomplicated DM patients, FGF-23 in DOP patients increased, while Irisin and N-MID decreased (P<0.05). According to ROC curve analysis, all three biomarkers demonstrated favorable predictive effects for DOP in DM patients (AUC=0.744-0.778). Through the subsequent logistic regression analysis, we confirmed that FGF-23 independently influenced DOP risk in DM, while Irisin and N-MID exerted protective effects (P<0.05, Table 2). After establishing the combined detection formula [1.083 + (-0.617xIrisin) + 0.113xFGF-23 + (-0.215 xN-MID)], the joint detection of Irisin, FGF-23, and N-MID was found to significantly enhance DOP diagnosis in DM patients, yielding 87.10% sensitivity, 74.58% specificity, and an AUC of 0.867 (P<0.001, Figure 1).

**Table 2:** Effects of Irisin, FGF-23, and N-MID on DOP. Note: regression coefficient (B), standard error (SE), odds ratio (OR), confidence interval (CI), fibroblast growth factor 23 (FGF-23), N-terminal propeptide of osteocalcin (N-MID).

	B	SE	OR	95%CI	Wals χ^2^	P
Irisin	-0.617	0.145	0.540	0.406-0.717	18.161	<0.001
FGF-23	0.113	0.031	1.120	1.054-1.191	13.262	<0.001
N-MID	-0.215	0.049	0.807	0.732-0.888	19.108	<0.001
constant	1.083	1.172	2.957	not reported	not reported	not reported

### Correlation of Irisin, FGF-23, and N-MID with DOP

**Figure 2:**
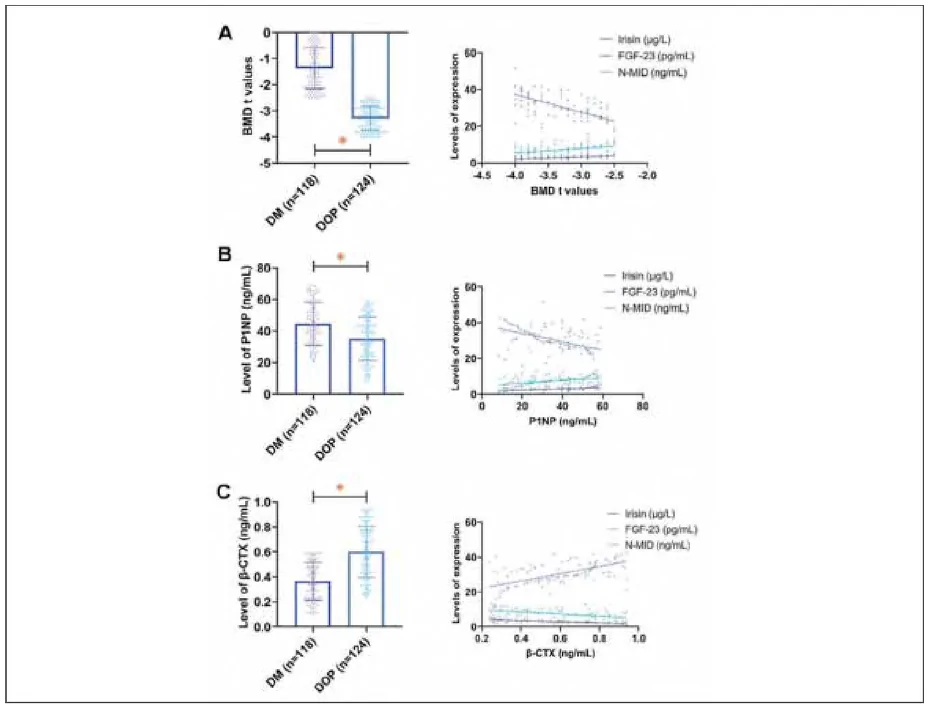
Correlation analysis of Irisin, FGF-23, N-MID and DOP. * indicates P < 0.05. (A) Comparison of BMD t values between DM patients and DOP patients, and correlation of BMD t values with Irisin, FGF-23, and N-MID in DOP patients. (B) Comparison of P1NP between DM patients and DOP patients, and correlation of P1NP with Irisin, FGF-23, and N-MID in DOP patients. (C) Comparison of β-CTX between DM patients and DOP patients, and correlation of β-CTX with Irisin, FGF-23, and N-MID in DOP patients.

BMD t value and P1NP level in DOP patients were lower than those in DM patients, while β-CTX was higher than that in DM patients (P<0.05). Pearson correlation analysis demonstrated a negative association between FGF-23 and BMD in DOP patients, in contrast to the positive correlations observed for Irisin and N-MID with BMD (P<0.05). Specifically, reduced BMD was associated with decreased Irisin and N-MID levels but elevated FGF-23. When examining markers related to bone formation and resorption, FGF-23 was found to correlate inversely with P1NP yet directly with β-CTX (P<0.05). In contrast, Irisin and N-MID correlated positively with P1NP and negatively with β-CTX (P<0.05, Figure 2, Table 3).

**Table 3:** Results of correlation analysis of Irisin, FGF-23, N-MID and DOP (r value). Note: fibroblast growth factor 23 (FGF-23), N-terminal propeptide of osteocalcin (N-MID), bone mineral density (BMD), N-terminal propeptide of type I collagen (PINP), b-crosslaps (β-CTX).

	BMD t values	P1NP	β-CTX
Irisin (μg/L)	0.713	-0.636	0.405
FGF-23 (pg/mL)	0.670	-0.470	0.394
N-MID (ng/mL)	-0.803	0.615	-0.512

### Variations in Irisin, FGF-23, and N-MID before and after treatment

Acarbose + sitagliptin treatment led to reduced FGF-23 in DOP patients, along with an increase in both Irisin and N-MID (P<0.05, Table 4). 

**Table 4:** Variations in Irisin, FGF-23, and N-MID before and after treatment. Note: fibroblast growth factor 23 (FGF-23), N-terminal propeptide of osteocalcin (N-MID).

	Before treatment	After treatment	t	P
Irisin (μg/L), (χ̄±s)	2.87±0.81	3.61±0.72	7.695	<0.001
FGF-23 (pg/mL), (χ̄±s)	30.43±6.92	26.01±4.38	6.006	<0.001
N-MID (ng/mL), (χ̄±s)	7.21±2.60	9.31±2.12	6.981	<0.001

### Prognostic significance of Irisin, FGF-23, and N-MID in DOP

**Figure 3:**
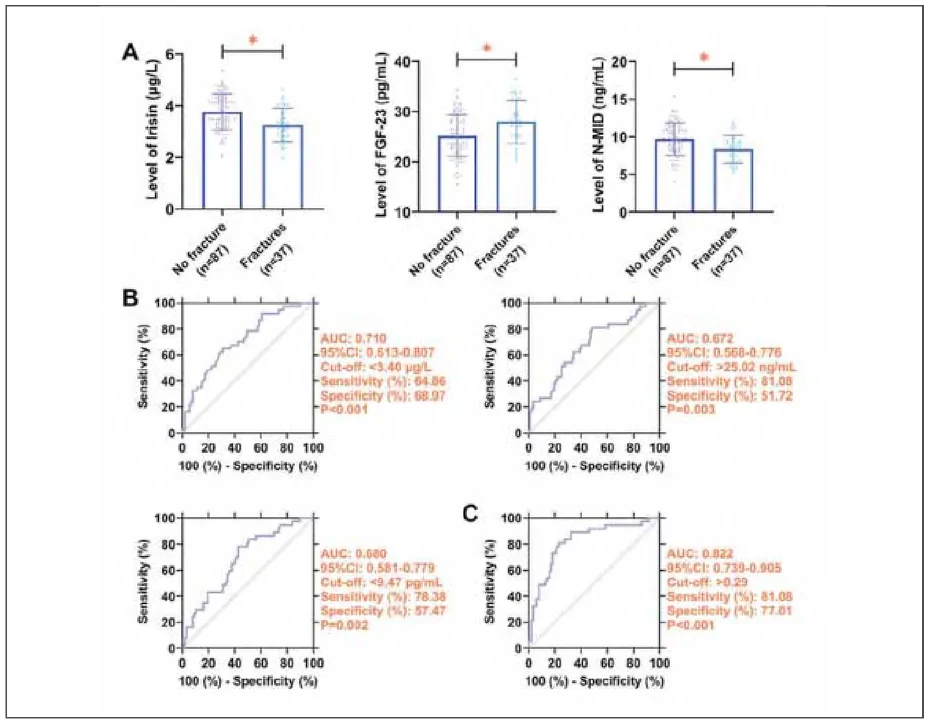
Correlation analysis of Irisin, FGF-23, N-MID and DOP. * indicates P < 0.05. (A) Comparison of BMD t values between DM patients and DOP patients, and correlation of BMD t values with Irisin, FGF-23, and N-MID in DOP patients. (B) Comparison of P1NP between DM patients and DOP patients, and correlation of P1NP with Irisin, FGF-23, and N-MID in DOP patients. (C) Comparison of β-CTX between DM patients and DOP patients, and correlation of β-CTX with Irisin, FGF-23, and N-MID in DOP patients.

During patient follow-up (duration >1 year), 37 cases developed osteoporotic fractures. The median follow-up was 12 [12][13] months. Fracture cases showed elevated FGF-23 and reduced Irisin/N-MID post-treatment versus non-fracture cases (P<0.05). ROC curve assessments revealed that Irisin, FGF-23, and N-MID each had a moderate prognostic value for fractures in DOP patients when used independently, with AUCs between 0.67 and 0.75. However, combining all three markers significantly [2.476 + (-1.207xIrisin) + 0.162xFGF-23 + (-0.379xN-MID)] improved predictive accuracy (AUC = 0.84; P<0.05, Table 5, Figure 3).

**Table 5:** Effect of Irisin, FGF-23, and N-MID on prognostic fracture in DOP patients. Note: regression coefficient (B), standard error (SE), odds ratio (OR), confidence interval (CI), fibroblast growth factor 23 (FGF-23), N-terminal propeptide of osteocalcin (N-MID).

	B	SE	OR	95%CI	Wals χ^2^	P
Irisin	-1.207	0.358	0.299	0.148-0.603	11.362	0.001
FGF-23	0.162	0.056	1.176	1.054-1.312	8.358	0.004
N-MID	-0.379	0.122	0.685	0.539-0.870	9.625	0.002
constant	2.476	2.230	11.891	not reported	not reported	not reported

## Discussion

This study marks the first systematic exploration into the dynamic regulatory effects of acarbose plus sitagliptin on Irisin, FGF-23, and N-MID among DOP patients. The findings not only confirm their significance in DOP but also provide a new biomarker panel for precise treatment, with great clinical implications for improving DOP patients' bone quality and decreasing their fracture susceptibility.

First of all, compared with uncomplicated DM individuals, FGF-23 was increased in DOP patients, while Irisin and N-MID were decreased, suggesting their possible involvement in DOP onset and progression. As has been well established, the pathological basis of DOP is linked to metabolic disturbances across multiple dimensions [4], with abnormally expressed Irisin, FGF-23, and N-MID persisting throughout the entire bone metabolic imbalance process. As a skeletal muscle-derived regulator of the muscle-bone axis, diminished Irisin levels serve as both an indicator of diminished muscle function and a direct cause of inhibited bone formation [12]. Research indicates that skeletal muscles regulate Irisin secretion through both mechanical stress and metabolic signals. In DOP patients, hyperglycemia-induced toxicities cause skeletal muscle atrophy and insulin resistance, both of which greatly suppress Irisin production [14]. Meanwhile, FGF-23 exerts its pathological role via the phosphorus metabolism-bone mineralization axis. Under a hyperglycemic-inflammatory milieu, osteocytes in DOP patients overproduce FGF-23. This, in turn, inhibits 1α-hydroxylase activity, reduces active vitamin D synthesis, impairs intestinal calcium absorption, and triggers secondary hyperparathyroidism, consequently establishing a vicious cycle of »elevated FGF-23—impaired bone mineralization« [15][13]. N-MID, an early sensitive marker of bone formation, directly reflects the activity of osteoblasts. In DOP patients, osteoclast activity enhancement coupled with osteoblast function suppression leads to reduced N-MID production, indicating an early-stage bone formation-absorption imbalance [16]. The close connection between the three factors (Irisin, FGF-23, and N-MID) with BMD, bone metabolism, and bone resorption further confirms their profound implications in DOP From a diagnostic perspective, combining Irisin, FGF-23, and N-MID measurements significantly enhanced early DOP identification, supporting their utility as a biomarker combination for DOP risk stratification. Notably, uncomplicated DM patients-despite insulin resistance-maintain higher Irisin than DOP cases (P<0.05), implicating skeletal muscle-bone axis dysfunction as a DOP-specific pathological feature [17].

On the other hand, our findings revealed a significant decrease in FGF-23 concentrations coupled with increased Irisin and N-MID levels in DOP patients following therapeutic intervention. It suggests that the acarbose-sitagliptin combination exerts a multi-dimensional synergistic effect on the regulation of Irisin, FGF-23, and N-MID. We believe that the increase in Irisin is strongly related to the improvement of skeletal muscle function. Acarbose mitigates glucotoxicity-induced muscle damage by reducing postprandial glycemic fluctuations, while sitagliptin enhances mitochondrial biosynthesis through AMPK pathway activation; together, these mechanisms enhance skeletal muscle's ability to secrete Irisin [18]. FGF-23 reduction reflects the rebalancing of phosphorus metabolism. Through DPP-4 inhibition, sitagliptin extends GLP-1's biological activity, which subsequently blocks urinary phosphate excretion by inhibiting renal tubular Na^+^-Pi cotransporters [19]. Furthermore, stabilized blood glucose levels may decrease inflammatory markers, possibly reducing their capacity to promote FGF-23 production in bone cells [20]. The early increase in N-MID indicates the rapid activation of osteoblast activity, which may be related to enhanced osteogenic differentiation by sitagliptin-induced Wnt/3-catenin pathway activation [21]. Of course, this view needs further study and confirmation. Regarding prognosis prediction, dynamic changes in Irisin, FGF-23, and N-MID were found to correlate intimately with a reduced fracture risk, which provides more intuitive clinical evidence and guidance for evaluating the prognosis of DOP patients in the future.

Based on the above findings, we suggest that a dynamic monitoring system integrating Irisin, FGF-23, and N-MID should be implemented in the clinical management of DM and DOP This strategy enables effective evaluation of the OP risk in DM patients and optimizes prognosis and rehabilitation predictions in DOP patients, thus improving long-term health outcomes. However, the applicability of our findings may be compromised by the small sample size and the single-center design. Besides, the absence of a sitagliptin-alone comparison group prevents a clear assessment of each drug's independent role. Moreover, further research is required to validate the sustained efficacy and safety profile of the combined treatment, with follow-up durations exceeding one year. Classical markers such as osteocalcin were not detected in this study, which may affect the comprehensivity of the association analysis between indicators. Finally, bone biopsy or high-resolution peripheral quantitative computerized tomography (HR-pQCT) could be employed in future research to evaluate bone microstructure alterations, so as to comprehensively quantify bone quality improvement.

## Conclusion

DOP patients present elevated FGF-23 while reduced Irisin and N-MID levels. Acarbose plus sitagliptin can significantly improve bone metabolism in DOP patients through a multidimensional regulatory mechanism involving "Irisin upregulation, FGF-23 downregulation, and N-MID elevation". Monitoring Irisin, FGF-23, and N-MID not only identifies new therapeutic targets for precise DOP treatment but also enables dynamic efficacy evaluation via early biomarker assessment.

### Availability of data and materials

The data that support the findings of this study are available from the corresponding author upon reasonable request.

### Funding

Not applicable.

### Acknowledgements

Not applicable.

### Conflict of interest statement

All the authors declare that they have no conflict of interest in this work.
